# Relationships between irregular work arrangements and occupational injuries in EU 27. Findings from the fifth European working condition survey

**DOI:** 10.1186/2049-3258-73-S1-P20

**Published:** 2015-09-17

**Authors:** Hanan Alali, Magd Abdel Wahab, Tanja Van Hecke, Bart De Clercq, Heidi Janssens, Lutgart Braeckman

**Affiliations:** 1 Gent university, Gent, Belgium; 2 Department of Mechanical Construction and Production, Ghent University, Gent, Belgium; 3 Department of Industrial Technology and Construction, Ghent University, Gent, Belgium; 4 Department of Public Health, Ghent University, Gent, Belgium

## Introduction

The associations between several measures of low employment quality and some specific health and safety outcomes have become the subject of more recent investigation. The main objective of our study is to examine the relationships between irregular work arrangements indicators including contract type, long working hours, multiple jobs, shift work, and occupational injuries, taking into account several sociodemographic and work characteristics.

## Methods

The study was based on the data of the fifth European working condition survey (EWCS), carried out by Eurofound from January to June 2010. For the purpose of this analysis, the analytical sample was restricted to a subgroup of 26.839 respondents from the 27 countries of the European Union, who were all workers with either a permanent contract, a temporary or a fixed contract. Associations between irregular work arrangements and occupational injuries were studied with multilevel modeling techniques.

## Results

About 9% of the workers suffered from an occupational injury over the past twelve months. An increased injury risk is observed for those working long hours (OR 1.24, 95% confidence interval 1.13 - 1.36), having multiple jobs (OR 1.25, 95% confidence interval 1.07 - 1.45) and shift work (OR 1.23, 95% confidence interval 1.09 - 1.38). However, the relationship between contract type and work injuries was not significant (OR 0.30, 95% confidence interval 0.79 - 1.07).

## Discussion

This study confirms that indicators of irregular work arrangements, with the exception of contract type, were significantly associated with occupational injuries. More attention should be paid to workers with low employment quality. Further efforts on the workplace, the organizational and political level are needed to avoid irregular work arrangements in order to improve workers' health and safety.

